# Investigating the Role Played by Osmotic Pressure Difference in Osmotic Dehydration: Interactions between Apple Slices and Binary and Multi-Component Osmotic Systems

**DOI:** 10.3390/foods12173179

**Published:** 2023-08-24

**Authors:** Xiaojuan Wang, Hao Feng

**Affiliations:** 1 Department of Agricultural and Biological Engineering, College of Agricultural, Consumer and Environmental Sciences, University of Illinois Urbana-Champaign, Urbana, IL 61801, USA; 2 Department of Food Science and Human Nutrition, College of Agricultural, Consumer and Environmental Sciences, University of Illinois Urbana-Champaign, Urbana, IL 61801, USA; 3 Department of Family and Consumer Sciences, North Carolina A&T State University, Greensboro, NC 27401, USA

**Keywords:** osmotic dehydration, mass transfer, osmotic pressure, synergistic effect

## Abstract

This study was performed to investigate a strategy to interpret the osmotic dehydration (OD) process through a focused exploration of osmotic pressure dynamics. The investigation first delved into the relationship between dehydration rate and the osmotic pressure difference between food and an osmotic solution. Apple slices was used as a model food material, and the OD process was conducted via sucrose, glucose, and maltose. The positive correlation between the osmotic pressure difference between food and osmotic solution and the dehydration rate suggested that this pressure difference served as the primary driving force for mass transfer within the OD process; for example, in 60% wt sucrose solution, the osmotic pressure of the solution decreased from 15.60 MPa to 12.98 MPa in the first 30 min, while the osmotic pressure of fresh apple slices increased from 1.49 MPa to 4.05 MPa; and this correlation between dehydration rate and osmotic pressure difference in product tissue and osmotic solution followed a linear relationship. Then, the study went further to investigate augmenting osmotic pressure of osmotic solution (sucrose and fructose) by adding auxiliary solutes (sodium chloride and calcium lactate). The results showcased that augmenting osmotic pressure within a sugar-based solution could be realized through the introduction of additive solutes, and what is more important is that this augmentation displayed a synergistic effect, which was more pronounced in solutions of lower sugar concentration. For example, the osmotic pressure of 45%wt fructose solution was 8.88 MPa, which could be increased to 10.05 MPa by adding 0.075% wt NaCl, while adding 0.075% wt NaCl to 59.14% wt fructose solution could increase osmotic pressure from 20.57 MPa to 21.22 MPa. In essence, this study proposed a strategic approach to studying the OD process by spotlighting osmotic pressure as a pivotal factor.

## 1. Introduction

Fruits and vegetables are a crucial part of the human diet due to their abundance of nutritional content, with components such as fiber, vitamins, minerals, antioxidants, and probiotics. However, the moisture content of fresh fruits and vegetables can be as high as 85% w.b., which makes them highly perishable. Drying is one effective strategy for prolonging their shelf life, reducing food waste, and promoting fruit and vegetable consumption, but nutrients lost in a high-temperature drying process is still a concern for high-quality food production [1]. Thus, the impregnation of variable nutrients into dried fruit and vegetable products, such as bioactive compounds [2,3], minerals [4,5], and prebiotic fibers [6,7], has been an active research area, especially with osmotic dehydration (OD) as a pretreatment and fortification step before finishing the drying processes (hot-air drying, microwave drying et al.) [8,9]. 

OD is performed by immersing a food material in a hypertonic solution (also called an osmotic solution). In an OD process, the osmotic pressure difference is considered as the driving force for mass transfer. In OD, the transport of water from a food material with a lower osmotic pressure to an osmotic solution having a higher osmotic pressure is the main mass transfer flux. Meanwhile, some soluble solids in osmotic solutions can migrate into food materials, and hence, the nutrition content of the food products can be changed [10,11,12,13].

The selection of osmotic solute is very important since it can affect not only the mass transfer rate, but also the quality of the final products. The most commonly used osmotic agents are sugars (sucrose, fructose, and glucose) for fruits and sodium chloride for vegetables and meats. The rate of solute transfer in an OD process is directly related to the concentration of osmotic solution and solute molecule size [14]. But low-molecular-weight sugars can accumulate in the sub-surface tissue or form a dense layer on the surface of food tissues, resulting in a new mass transfer barrier. Salt molecules can penetrate into the food tissue to a much greater depth than can sugars. Thus, a multiple-solute osmotic solution might be preferable for promoting the process [15]. It was reported that adding calcium salt to sucrose solutions in OD not only speeds up the dehydration rate, but also changes the flavor of the final products and increases the products’ firmness [16].

Many previous studies have focused on the effects of the type and concentration of solute in an osmotic solution on mass transfer in OD process, but little attention has been given to the osmotic pressure of OD systems, or the role played by osmotic pressure in mass transfer. During an OD process, as water migrates from food into an osmotic solution and solute of osmotic solution enters the food, the osmotic pressure of the surrounding osmotic solution and that of the osmotic pressure of the liquid inside the food matrix (inside cellulose space or inside plant cells) are changing constantly. The dynamic changes in osmotic pressure determine the rate of the multi-component and two-directional mass transfer taking place in OD processes, and hence, the subsequent changes in the drying performance and product quality [2,15]. Till now, very little is known about the dynamics of the osmotic pressure and its role in an OD process, because they have not been previously the focus of study. In addition, very few studies have attempted to determine the osmotic pressure of multiple-solute osmotic solutions [16].

The purpose of this study was to investigate the role of osmotic pressure in osmotic dehydration and provide new insights for the design and control of OD processes by formulating osmotic solution. To achieve this purpose, experiments were carried out in two stages: 1. studying the role played by the osmotic pressure difference between the food materials and that of the osmotic solution on mass transfer in OD process, with apple being used as the model food for the purpose, and sucrose, glucose, and maltose being used as osmotic agents; 2. evaluating the change in osmotic pressure in multiple-solute osmotic solutions, which were formed by the most commonly used osmotic agents of sucrose and fructose, as well as two typical food ingredients, including sodium chloride (NaCl), which is considered to have a good penetration into food materials in the OD process [15], and calcium lactate (CaLac), which is often used for mineral impregnation in the OD process [17,18]. 

## 2. Materials and Methods

### 2.1. Osmotic Dehydration Experiments

‘Fuji’ apples (Malus domestica Borkh) were purchased at a local grocery store, stored in a cold room at 1 °C, and used within one week. The apples were first cut into slices of 5 mm thickness with a slicer (Mliter, 150 W Electric Professional, Shenzhen, China). Then, a corer (14 mm diameter) was used to obtain disk samples (5 mm thickness and 14 mm diameter) from the slices and each disk weighed 0.937 ± 0.038 g.

Sucrose, glucose, fructose, and maltose were purchased from Fisher Scientific (Fisher, Waltham, MA, USA). Sucrose solution was prepared at 40% wt, 50% wt, and 60% wt, and glucose, fructose, and maltose solutions were prepared at 50% wt. 

Osmotic dehydration was performed in an incubator shaker (I 24R Incubator shaker, New Brunswick Scientific Co., Inc., Edison, NJ, USA) at 40 °C, 200 rpm, and 1 atm [2,15]. Five apple slices with a total mass of 4.60 ± 0.115 g were placed in a 120 mL glass vessel with a lid (Qorpak™, Clinton, PA, USA). Different osmotic solutions (50 mL each) were added to the glass vessels to reach an apple to osmotic solution ratio of 1:10 *w*/*v*, which was large enough to minimize the effect due to changes in solution concentration during OD [15]. At the selected times (0.5 h, 1.0 h, 1.5 h, and 2.0 h), one glass vessel was removed from the shaker, and all the five apple slices in the vessel were removed from the osmotic solution, gently blotted to remove the solution on the surface, and weighted. The OD tests were terminated after 2 h because the water loss became insignificant thereafter. All the experiments were conducted in triplicate. 

### 2.2. Osmotic Pressure in Multiple Solutes System

Sucrose, fructose, sodium chloride (NaCl), and calcium lactate (CaLac) were purchased from Fisher Scientific (Fisher, Waltham, MA, USA). Different concentrations of binary, ternary, and quaternary solutions were prepared for water activity measurement, and the choices of solute concentrations were determined according to the central composite design in the response surface method (RSM) using Design-Expert (Free trial, Stat-Ease, Inc., Minneapolis, MN, USA). For sucrose and fructose, the concentration lower and upper limit levels were 35% wt and 55% wt, which were chosen according to the concentration of the most commonly used osmotic solutions [15]. The concentration lower and upper limit levels of NaCl were set at 0.05% wt and 0.1% wt. The concentration lower and upper limit levels of CaLac were set at 1% wt and 2% wt. The concentration levels of both salts were determined according to their concentration in commercial beverage products [18]. The concentration limit levels were applied as the input in RSM for generating concentration values in the following tests. The solutes were weighed using an analytical balance with a precision of 0.1 mg (AP210S, UHAUS, Parsippany, NJ, USA) and dissolved in distilled water. The studied osmotic systems are summarized in Table 1. The solutions were kept at 40 °C in accordance with the most common temperature in OD process. 

### 2.3. Water Activity 

Water activity ($a_{w}$) of apples and osmotic solutions was measured using a water activity meter (AQUALAB 4TE, METER Group, Inc., Pullman, WA, USA) with the chamber temperature set at 40 °C, the same as the processing temperature of OD process. For liquid samples, the $a_{w}$ can be measured directly by pouring the sample into the sample pan of the water activity meter. For apple samples, the samples were chopped into small chips with a kitchen knife, and the chips were spread evenly in the sample dish. All the tests were conducted with triplicate samples, and triplicate tests were conducted on each sample.

### 2.4. Mass Transfer in Osmotic Dehydration

The moisture contents of the fresh and treated apples were determined by drying at 70 °C and 25-inch Hg vacuum pressure in a vacuum oven (Isotemp Vacuum Oven Model 281 A, Fisher, Waltham, MA, USA) for 24 h [19]. Water loss (WL) is the net water loss in fresh samples (g water/g dry solid) after a certain time of osmotic treatment, and it can be calculated by the following equation:

(1) $$WL\left(t\right)=\frac{W_{0}X_{0}-W_{t}X_{t}}{W_{0}\left(1-X_{0}\right)}$$

where W and X are the weights of apple slices and moisture content in wet basis, respectively. The subscripts 0 and *t* indicate the weight and moisture content of apple slices after 0 h and *t* h osmotic dehydration, respectively. Solid gain (g solute/g dry solid) is the weight of the solutes that are absorbed into apple slices during osmotic dehydration. It can be expressed as:

(2) $$SG\left(t\right)=\frac{W_{t}(1-X_{t})-W_{0}(1-X_{0})}{W_{0}\left(1-X_{0}\right)}$$

Dehydration rate (g water/g dry weight/min) was calculated from WL results with the following equations:

(3) $$WL\;rate=\frac{{WL}_{t_{2}}-{WL}_{t_{1}}}{t_{2}-t_{1}}$$

where, $t_{1}$ and $t_{2}$ were two consecutive sampling time, and ${WL}_{t_{1}}$ and ${WL}_{t_{2}}$ were water loss at $t_{1}$ and $t_{2}$, respectively. 

### 2.5. Osmotic Pressure

Osmotic pressure is defined as the difference of the pressure in two systems in equilibrium, one of which is often pure water, and under such a condition, the chemical potentials of the two systems are equal. The equilibrium of two systems defined by chemical potentials is shown in Equation (4).

(4) $$\mu_{0}^{i}+{\bar{V}}_{w}p^{i}+RTln\left(a_{w}^{i}\right)=\mu_{0}^{j}+{\bar{V}}_{w}p^{j}+RTln{(a}_{w}^{j})$$

where $\mu_{0}^{i}$ and $\mu_{0}^{j}$ is the standard chemical potentials of water in system *i* and *j*, respectively, which can be considered equal [20], ${\bar{V}}_{w}$ is the partial molal volume of pure water (${\bar{V}}_{w}$ = 0.018 L/mol), p is the hydrostatic pressure, R is the gas constant, and $a_{w}$ is the water activity. When system *i* is pure water, and system *j* is either osmotic solution or apple tissue, the osmotic pressure of the osmotic solution and apple tissue can be given by Equations (7) and (6) (note $a_{w}^{i}=1$):

(5) $$\pi^{s}=-\frac{RT\;}{{\bar{V}}_{w}}\;ln\left(a_{w}^{s}\right)$$

(6) $$\pi^{t}=-\frac{RT\;}{{\bar{V}}_{w}}\;ln\left(a_{w}^{t}\right)$$

where superscripts t and s represent water in apple tissue and osmotic solution, respectively. Thus, the osmotic pressure difference π between the apple tissue and the osmotic solution can be written as:

(7) $$\Delta \pi =\pi^{s}-\pi^{t}=-\frac{RT}{{\bar{V}}_{w}}\;ln(\frac{a_{w}^{s}}{a_{w}^{t}})\;$$

It was pointed out in 1894 that osmotic pressure is not a function of the relationship of solute particles to solvent particles, but a function of the mole fraction of the solvent present [19]. For dilute solutions, they can be considered as ideal solutions, and they will obey Raoult’s law, in which the partial vapor pressures are proportional to the mole fractions. Thus, the water activity can be substituted with the mole fraction of the solvent x in Equation (5).

(8) $$\pi =-\frac{RT}{{\bar{V}}_{w}}\;ln(x)$$

The correlation of dehydration rate or solid gain and the π between apple tissue and osmotic solutions were analyzed using the ORIGIN2021 software (Origin 2021b, OriginLab Corporation, Northampton, MA, USA). The correlation between osmotic pressure and solution concentration was also analyzed. 

Hamdan et al. proposed the concept of synergistic factor (SF) to study the synergistic effect of a second solute on the osmotic pressure of ternary solutions [16].

(9) $$SF=\pi_{ternary}-\left(\pi_{1}+\pi_{2}\right)$$

where $\pi_{ternary}$, $\pi_{1},$ and $\pi_{2}$ are the osmotic pressure of the ternary system, the osmotic pressure of solute 1 solution, and the osmotic pressure of solute 2 solution at corresponding concentrations, respectively. At SF=0, the solution is an additive system. At SF>0, the solution is a positive synergy system. At SF<0, the solution is a negative synergy system.

Equations (5) and (8) were used for the osmotic pressure calculation of osmotic solutions in the study of Section 2.1 and Section 2.2. Equation (6) was used for the osmotic pressure calculation of apple tissues in the study of Section 2.1. Equation (7) was used to calculate the osmotic pressure difference between apple tissue and osmotic solution in the study of Section 2.1.

## 3. Results and Discussion

### 3.1. Osmotic Pressure in OD Process

**Figure 1:**
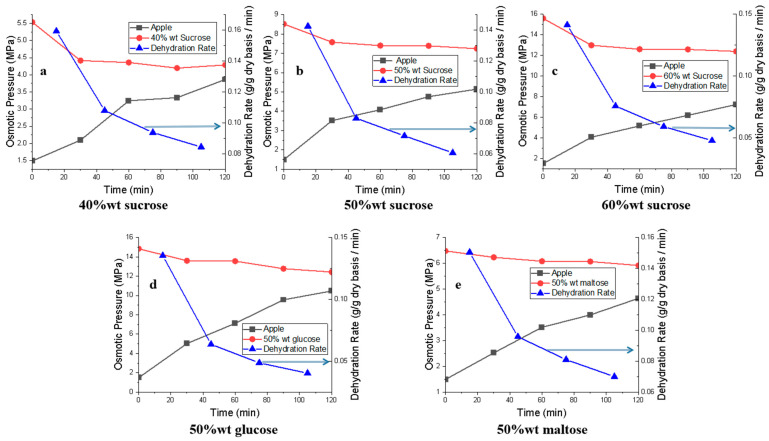
Changes in dehydration rate and osmotic pressure as a function of osmotic presume in apple tissue and different solutions in OD process.

Figure 1 shows the osmotic pressures of both the apple tissue and sugar solutions, as well as the dehydration rate of OD as a function of time. A decrease in the osmotic pressure of the sugar solutions can be observed for all three sugars. For example, in 60%wt sucrose solution, the osmotic pressure of the solution decreased from 15.60 MPa to 12.98 MPa in the first 30 min, and it decreased from 12.98 MPa to 12.39 MPa in the following 1.5 h. On the other hand, the osmotic pressures of apple tissues increased in all OD experiments. For example, the osmotic pressure of fresh apple was 1.49 MPa, and its osmotic pressure increased to 4.05 MPa and 7.22 MPa after treatment with 60% wt sucrose solution for 0.5 h and 2.0 h, respectively. It is because that water transfers from apple to sugar solution in OD process, which changes the water activity in both sides. The dehydration rate was high at the beginning of the OD experiments, and it decreased as OD proceeded. It can also be seen that the dehydration rate in OD tests with a higher sugar solution concentration is lower than that with a higher sugar solution. The dehydration rate one hour after the start of OD was 0.09, 0.07, and 0.06 g/g dry basis per min in sucrose solutions of 40, 50, and 60% wt, respectively. Lazarides et al. assumed that OD at a high sugar concentration could promote the formation of concentrated subsurface solid layers that would affect the osmotic difference across the interface of food material and solution [21]. Indeed, the formation of a solid layer on the surface of food would help to slow down the mass transfer, thus reducing the osmotic pressure difference. Other researchers also held that an increase in sugar concentration could accelerate the absorption of sugar on the surface of food materials, thus decreasing the mass exchange between food material and solution [22]. Similarly, the reduced dehydration rate recorded in this study when the sugar concentration was high in the OD solution could be attributed to a concentration-induced surface layer when the sugar concentration, which could impart increased resistance to mass transfer. The effect of water activity on an OD process can be estimated using the osmotic pressure values using Equation (5).

As shown in Figure 1b,d,e, the initial osmotic pressures of the solutions were 8.52 MPa, 14.84 MPa, and 6.48 MPa in 50% wt sucrose, 50% wt glucose, and 50% wt maltose solutions, respectively. Glucose is a simple sugar with a smaller molecule weight. As a result, a given unit mass of sugar solution contains a higher number of molecules compared to other types of sugar molecules. This abundance of glucose molecules could contribute to an elevated osmotic pressure within the solution [22]. The difference in the osmotic pressures of 50%wt sucrose and 50%wt maltose solutions might be caused by the different simple sugar molecules in their molecule formation. The sucrose molecule has one glucose molecule and one fructose molecule, while the maltose molecule has two glucose molecules. 

**Figure 2:**
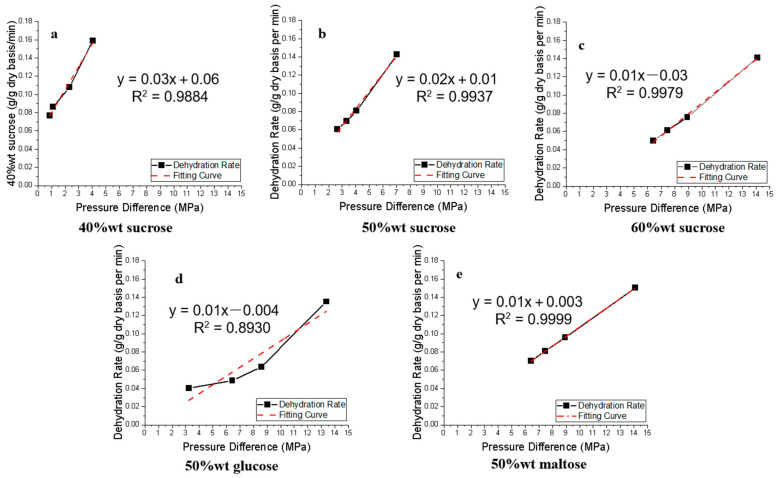
Correlation between dehydration rate and osmotic pressure difference between apple tissue and osmotic solution in OD process.

Figure 2 shows the relationship between the dehydration rate and the osmotic pressure difference between the apple tissue and the osmotic solution, together with the linear regression equations. All curves have exhibited some tendency to be concave upwards, and it is more pronounced for the glucose solution, which results in a relatively poor linear correlation in glucose solution. This is an observation that deserves further study to understand the interactions between the osmotic pressure and dehydration rate when the sugars have different molecular structures. An important observation is that the osmotic difference is positively correlated to the OD dehydration rate. This indicates that the osmotic difference functions as the mass transfer driving force in an OD process. The slope of correlation equations indicates the increase in dehydration rate is caused by a unit osmotic pressure difference. It can be observed that increasing sucrose concentration diminishes the effect of osmotic pressure difference on the dehydration rate. For instance, in 60% wt sucrose solution, when the osmotic pressure difference between apple and solution increased 1 MPa, the dehydration rate increased 0.01 g/g dry basis per min (Figure 2c), while in 40% wt sucrose solution, an increase of 1 MPa in osmotic pressure difference led to an increase in dehydration rate by 0.03 g/g dry basis per min. 

**Figure 3:**
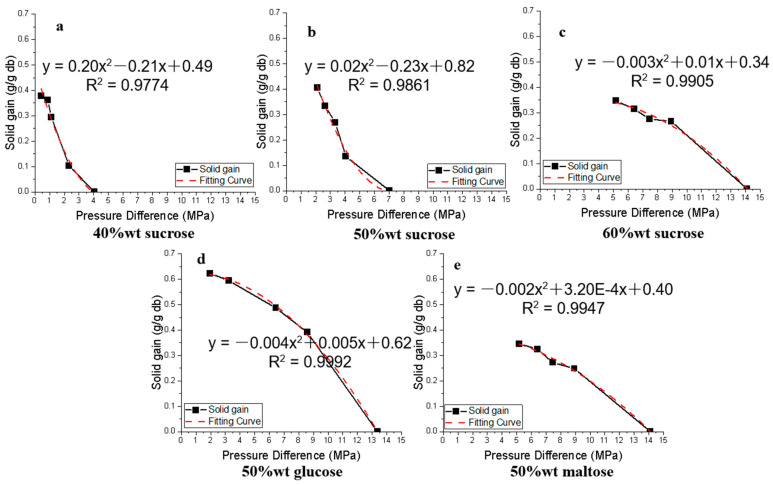
Correlation of solid gain and osmotic pressure difference between apple and sucrose solutions of different concentrations and glucose and maltose solutions.

The amount of solid gain as a function of osmotic pressure difference between that of apple tissue and the different osmotic solutions in the OD process is given in Figure 3. A second-order nonlinearity regression was found to be a good fit for the solid gain data (R^2^ > 0.9774). Overall, the accumulated solid gain is negatively correlated to osmotic pressure difference. The intercept of the correlation equations indicates the possible solid gain in apples that could be obtained when the OD process reaches equilibrium. In other words, it is the possible amount of solute that could be impregnated into apple tissues, when the osmotic pressure difference approaches zero.

### 3.2. Osmotic Pressure in Binary Systems: One Sugar/One Salt + Water

**Figure 4:**
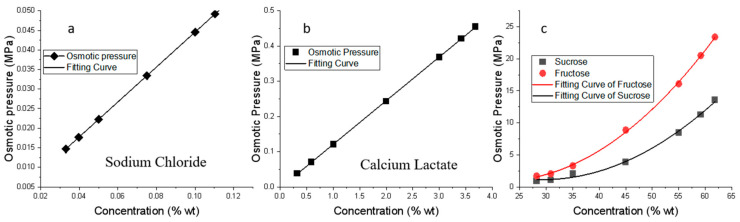
Effect of salt or sugar concentration on osmotic pressure in selected binary systems at 40 °C.

The osmotic pressures of four binary systems, e.g., sucrose/water, fructose/water, sodium chloride/water, and calcium lactate/water, as a function of solute concentration are shown in Figure 4. The two electrolyte systems with salts exhibited a good linear relationship, while the two non-electrolyte systems can be well-described by a secondary-order non-linear regression equation, as shown in Table 2. At the same concentration, the osmotic pressure of fructose was higher than that of sucrose (Figure 4c), meaning that fructose-rich systems, such as fruit juices may be good for OD applications. 

For the electrolytes, the dilute solutions were considered as an ideal solution, the osmotic pressure was calculated by Equation (8), and the obtained values were in agreement with those from previous studies [16]. The slopes of the fitting equations in Table 2 indicate that the osmotic pressure in the sodium chloride solution increases 0.45/0.12 = 3.75 times faster than that of the calcium lactate system. It is noted that the concentration ranges for th two salts are quite different with the calcium lactate being at a much higher concentration than sodium chloride. 

### 3.3. Osmotic Pressure in Ternary Systems: One Sugar + One Salt + Water

**Figure 5:**
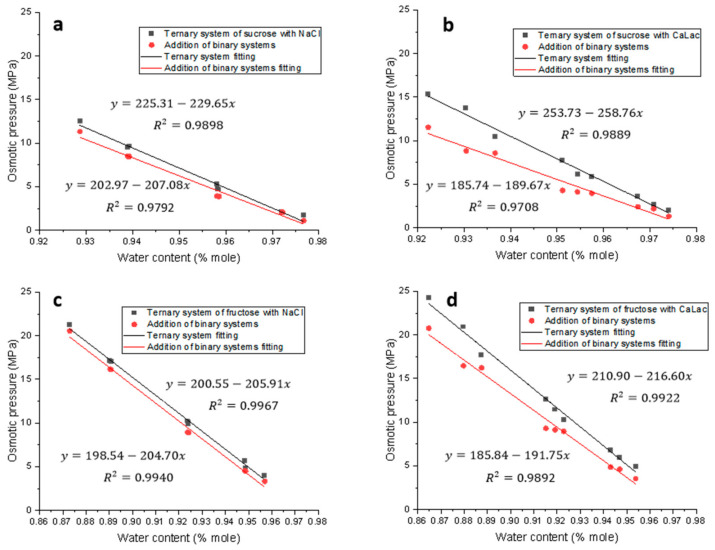
Effect of water content on osmotic pressure in sucrose and fructose ternary systems at 40 °C. (**a**) Sucrose ternary system with NaCl; (**b**) sucrose ternary system with CaLac; (**c**) fructose ternary system with NaCl; (**d**) fructose ternary system with CaLac. Osmotic pressure addition of binary systems was the sum of osmotic pressures generated by the binary system formed with each individual solute at the same concentration as in ternary systems.

The detailed information on osmotic pressures in the studied four ternary systems is summarized in Table 3, Table 4, Table 5 and Table 6, while the effect of water content on osmotic pressure in the systems is shown in Figure 5.

In all the ternary systems, the addition of a second solute increased the osmotic pressure of the mixed system ($\pi_{mix}$). The $\pi_{mix}$ value was higher than that of the individual binary systems formed with the same solute at the same concentration, as shown in Table 3, Table 4, Table 5 and Table 6. For example, in the ternary system of sucrose-NaCl-water, at a fixed concentration of sucrose, increasing the concentration of NaCl resulted in an increase in the osmotic pressure of the system ($\pi_{mix}$) (Table 3). When the concentration of sucrose was 45% wt, increasing NaCl concentration from 0.04 to 0.110%wt resulted in an increase in the synergistic factor (SF) of nearly 1.7-fold. However, at a higher sucrose solution, e.g., ≥55% wt, there was no synergistic effect. Similar results can be observed in the ternary systems of fructose and NaCl. This may be caused by the difference in viscosity and density of the sucrose and fructose solutions. At 40 °C, the viscosity of 55% wt sucrose solution is higher than that of 55% wt fructose solution [23]. High viscosity may limit the effect of Na^+^ and Cl^−^ on the freedom of water molecules and their hydrogen bonds. When CaLac was used as a second solute to the sugar solutions, for both sucrose and fructose, the osmotic pressure of the ternary systems and the synergistic effects were increased when increasing the salt concentration at all three sugar concentration levels (35%, 45%, and 55% wt). 

In Figure 5, it can be seen that the synergistic effect (SF) decreases as the water content increases in all ternary systems. For sucrose solutions, with the increasing water content, the synergistic effect decreased faster in the sucrose + CaLac + water system (Figure 5b) than that in sucrose + NaCl + water (Figure 5a). In fructose solutions, with NaCl as a second solute, the synergistic effect decreased slightly as water content increased (Figure 5c). Adding CaLac to fructose solutions, it could be seen that water content had a greater impact on synergistic effect than that with NaCl. In all, water content had a greater impact on the synergistic effect in sucrose solutions than that in fructose solutions. The reason might be due to the different structures of sucrose and fructose, as well as the interactions when an additional solute was added into the OD solution. 

### 3.4. Osmotic Pressure in Quaternary Systems: One Sugar + Two Salt + Water

**Figure 6:**
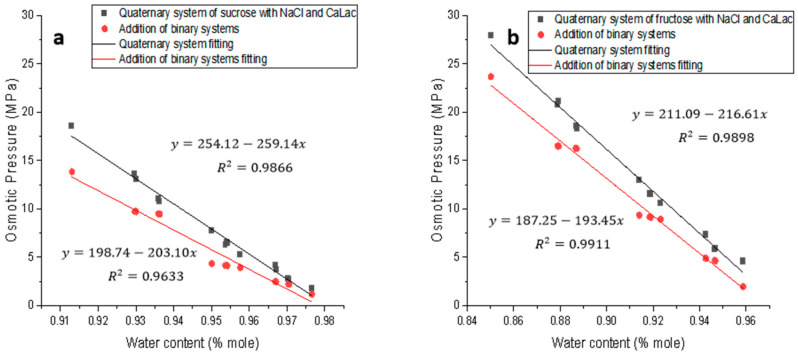
Effect of water content on osmotic pressure synergistic effect in sucrose and fructose quaternary system at 40 °C. (**a**): Sucrose quaternary system with NaCl and CaLac; (**b**): fructose quaternary system with NaCl and CaLac; osmotic pressure addition of binary systems was the sum of osmotic pressures generated by the binary system formed with each individual solute at the same concentration as in ternary systems.

The values of osmotic pressure terms of two quaternary systems (sucrose + NaCl + CaLac + water and fructose + NaCl + CaLac + water) are summarized in Table 7 and Table 8, while the effect of water content on osmotic pressure in the systems is shown in Figure 6. In both systems, at a fixed concentration of sugar and one salt, the osmotic pressure of the quaternary system and the synergistic effect increased as another salt concentration increased. At the same sugar and salt concentration, the synergistic effect (SF) in fructose solutions was more pronounced than those in sucrose solutions. This could be also linked to the difference in molecular structures of sucrose and fructose, e.g., sucrose has higher polarity than fructose, resulting in reduced chemical potentials in the solution. The osmotic pressures again exhibited a linear relationship with water content in both sugar systems (Figure 6). The more dilute the solution was, the smaller the synergistic effect was. In addition, water content had more effect on the synergistic effect in sucrose solutions than that in fructose solutions, similarly to that seen in the ternary systems. 

In general, considering osmotic dehydration as a dynamic process, in which all the components in the food and in the osmotic solution are constantly changing, a good understanding of the root cause of these changes is critical for process design and process control purposes. In this study, a fundamental approach is taken to provide insights on how the changes in the concentration of solutes in osmotic solution will impact the mass transfer process. In the analysis, a new concept, the osmotic pressure difference, was proposed as the driving force for mass transport in osmotic dehydration, whose role has been well demonstrated by dehydration rate and solid gain rate (Figure 1, Figure 2 and Figure 3). With this understanding, if the objective function in the optimization of osmotic process is to minimize the total dehydration time, for instance, in operation, we should make an effort to maintain the concentration of sugar at a certain level in order to maintain a relatively large osmotic pressure difference. Periodically supplementing the osmotic solution with sugar will be necessary. Similarly, if the purpose of OD is to fortify the food by infusion of certain minerals, then careful control of the concentration of the target mineral in the osmotic solution becomes important. In both cases, a detailed solute concentration change kinetic study needs to be performed for a specific food type, and the effect of other solutes, such as NaCl, needs to be considered, as all real foods are multi-component systems. 

## 4. Conclusions

This study investigated the role played by osmotic pressure difference between the food sample and the osmotic solution. The osmotic pressure difference is shown to be positively correlated to the OD de-watering rate; thus, it can be considered as the driving force for mass transfer in OD processes. The relationship between the dehydration rate and osmotic pressure difference of product tissue and osmotic solution followed a linear relationship. However, the data on the relation between the solid-gain and osmotic pressure difference of apple tissue and osmotic solutions fit well to quadratic polynomial equations. The estimation of osmotic pressure difference was based on measured and calculated values of sugar solutions, as well as the osmatic pressure when a salt (NaCl or CaLac) was added to a sugar solution. The selection of osmotic agents can affect the efficiency of the osmotic dehydration process and the nutrition profile of the final products. For the systems studied, fructose solutions had a higher osmotic pressure than those of sucrose at the same sugar concentration, which would provide more driving force for mass transfer in OD processes. Adding a second or a third solute to the sugar solutions results in an increase in the osmotic pressure of the final multi-solute solution. The increase caused by adding individual solutes was found to be synergistic in all the systems studied. Moreover, the concept of osmotic pressure difference may provide a useful tool for the design and optimization of an OD process by controlling the concentration of sugar or salt during an OD process. These findings will provide insight into the design of osmotic solutions that can achieve enhanced dewatering rate with less solute addition. Future study is needed to design and validate the use of osmotic pressure difference as a tool to optimize an OD process for nutrient fortification applications.

## Figures and Tables

**Table 1:** Osmotic systems investigated in this work.

Osmotic System	Osmotic Solution
Binary	Sucrose + water
	Fructose + water
	NaCl + water
	CaLac + water
Ternary	Sucrose + NaCl + water
	Sucrose + CaLac + water
	Fructose + NaCl + water
	Fructose + CaLac + water
Quaternary	Sucrose + NaCl + CaLac + water
	Fructose + NaCl + CaLac + water

**Table 2:** Fitting equation of osmotic pressure and solute concentration in selected binary systems.

Solute	Fitting Equation	R^2^
Sucrose	$y=0.11x^{2}-0.67x+10.9155$	0.9968
Fructose	$y=0.01x^{2}-0.57x+7.75$	0.9980
Sodium Chloride	y=0.45x−0.000017	1
Calcium Lactate	y=0.12x−0.0028	0.9999

**Table 3:** Synergistic effect comparison between binary and ternary sucrose systems with the NaCl additive.

Concentration (% wt)		Osmotic Pressure (MPa)				Synergistic Factor (MPa) $SF=\pi_{mix}-\pi_{addition}$
Sucrose	NaCl	$\pi_{sucrose}$	$\pi_{NaCl}$	$\pi_{addition}$	$\pi_{mix}$	
30.858	0.075	1.069	0.033	1.102	1.701	0.599
35.000	0.050	2.043	0.022	2.065	2.077	0.012
35.000	0.100	2.043	0.045	2.088	2.136	0.048
45.000	0.040	3.868	0.018	3.885	4.677	0.791
45.000	0.075	3.868	0.033	3.901	4.736	0.835
45.000	0.110	3.868	0.049	3.917	5.250	1.333
55.000	0.050	8.448	0.022	8.471	9.649	1.178
55.000	0.100	8.448	0.045	8.493	9.541	1.048
59.142	0.075	11.286	0.033	11.319	12.521	1.201

$\pi_{mix}=\pi_{sucrose+NaCl}$; $\pi_{addition}=\pi_{sucrose}+\pi_{NaCl}.$

**Table 4:** Synergistic effect comparison between binary and ternary systems of sucrose with the additive of CaLac.

Concentration (% wt)		Osmotic Pressure (MPa)				Synergistic Factor (MPa) $SF=\pi_{mix}-\pi_{addition}$
Sucrose	CaLac	$\pi_{sucrose}$	$\pi_{CaLac}$	$\pi_{addition}$	$\pi_{mix}$	
30.858	2.000	1.069	0.243	1.312	1.984	0.672
35.000	1.000	2.077	0.120	2.198	2.636	0.438
35.000	3.000	2.043	0.368	2.411	3.591	1.180
45.000	0.586	3.868	0.070	3.938	5.871	1.933
45.000	2.000	3.868	0.243	4.111	6.102	1.991
45.000	3.414	3.868	0.421	4.288	7.710	3.421
55.000	1.000	8.448	0.120	8.569	10.444	1.876
55.000	3.000	8.448	0.368	8.817	13.697	4.881
59.142	2.000	11.286	0.243	11.529	15.349	3.820

$\pi_{mix}=\pi_{sucrose+CaLac}$; $\pi_{addition}=\pi_{sucrose}+\pi_{CaLac}.$

**Table 5:** Synergistic effect comparison between binary and ternary systems of fructose with the additive of NaCl.

Concentration (% wt)		Osmotic Pressure (MPa)				Synergistic Factor (MPa) $SF=\pi_{mix}-\pi_{addition}$
Fructose	NaCl	$\pi_{fructose}$	$\pi_{NaCl}$	$\pi_{addition}$	$\pi_{mix}$	
30.858	0.075	3.295	0.033	3.328	3.982	0.653
35.000	0.050	4.493	0.022	4.515	4.831	0.316
35.000	0.100	4.493	0.045	4.537	5.646	1.108
45.000	0.040	8.878	0.018	8.896	9.840	0.944
45.000	0.075	8.878	0.033	8.912	10.051	1.140
45.000	0.110	8.878	0.049	8.927	10.217	1.289
55.000	0.050	16.101	0.022	16.123	17.041	0.918
55.000	0.100	16.101	0.045	16.146	17.106	0.961
59.142	0.075	20.526	0.033	20.559	21.222	0.662

$\pi_{mix}=\pi_{fructose+NaCl}$; $\pi_{addition}=\pi_{fructose}+\pi_{NaCl}.$

**Table 6:** Synergistic effect comparison between binary and ternary systems of fructose with the additive of CaLac.

Concentration (% wt)		Osmotic Pressure (MPa)				Synergistic Factor (MPa) $SF=\pi_{mix}-\pi_{addition}$
Fructose	CaLac	$\pi_{fructose}$	$\pi_{CaLac}$	$\pi_{addition}$	$\pi_{mix}$	
30.858	2.000	3.295	0.243	3.538	4.906	1.368
35.000	1.000	4.493	0.120	4.613	5.946	1.333
35.000	3.000	4.493	0.368	4.861	6.787	1.927
45.000	0.586	8.878	0.070	8.948	10.253	1.304
45.000	2.000	8.878	0.243	9.121	11.432	2.311
45.000	3.414	8.878	0.421	9.299	12.610	3.311
55.000	1.000	16.101	0.120	16.221	17.661	1.439
55.000	3.000	16.101	0.368	16.469	20.926	4.457
59.142	2.000	20.526	0.243	20.769	24.250	3.481

$\pi_{mix}=\pi_{fructose+CaLac}$; $\pi_{addition}=\pi_{fructose}+\pi_{CaLac}.$

**Table 7:** Synergistic effect comparison between binary and quaternary systems of sucrose with the additives of NaCl and CaLac.

Concentration (% wt)			Osmotic Pressure (MPa)					Synergistic Factor (MPa) $SF=\pi_{mix}-\pi_{addition}$
Sucrose	CaLac	Na	$\pi_{sucrose}$	$\pi_{CaL}$	$\pi_{NaCl}$	$\pi_{addition}$	$\pi_{mix}$	
28.182	2.000	0.075	0.909	0.243	0.033	1.185	1.794	0.609
35.000	1.000	0.050	2.077	0.120	0.022	2.220	2.734	0.514
35.000	1.000	0.100	2.077	0.120	0.045	2.242	2.837	0.595
35.000	3.000	0.050	2.077	0.368	0.022	2.468	3.734	1.266
35.000	3.000	0.100	2.077	0.368	0.045	2.490	4.165	1.675
45.000	0.318	0.075	3.868	0.038	0.033	3.939	5.295	1.356
45.000	2.000	0.033	3.868	0.243	0.015	4.125	6.490	2.364
45.000	2.000	0.075	3.868	0.243	0.033	4.144	6.586	2.441
45.000	2.000	0.117	3.868	0.243	0.052	4.163	6.324	2.161
45.000	3.682	0.075	3.868	0.455	0.033	4.356	7.715	3.359
55.000	1.000	0.050	9.330	0.120	0.022	9.472	10.755	1.283
55.000	1.000	0.100	9.330	0.120	0.045	9.495	11.046	1.552
55.000	3.000	0.050	9.330	0.368	0.022	9.720	13.068	3.348
55.000	3.000	0.100	9.330	0.368	0.045	9.743	13.665	3.923
61.818	2.000	0.075	13.565	0.243	0.033	13.841	18.584	4.743

$\pi_{mix}=\pi_{sucrose+CaLac+NaCl}$; $\pi_{addition}=\pi_{sucrose}+\pi_{CaLac}+\pi_{NaCl}.$

**Table 8:** Synergistic effect comparison between binary and quaternary systems of fructose with the additives of NaCl and CaLac.

Concentration (% wt)			Osmotic Pressure (MPa)					Synergistic Factor (MPa) $SF=\pi_{mix}-\pi_{addition}$
Fructose	CaLac	Na	$\pi_{fructose}$	$\pi_{CaLac}$	$\pi_{NaCl}$	$\pi_{addition}$	$\pi_{mix}$	
28.182	2.000	0.075	1.706	0.243	0.033	1.983	4.597	2.614
35.000	1.000	0.050	4.493	0.120	0.022	4.635	5.951	1.316
35.000	1.000	0.100	4.493	0.120	0.045	4.658	5.856	1.199
35.000	3.000	0.050	4.493	0.368	0.022	4.483	7.268	2.385
35.000	3.000	0.100	4.493	0.368	0.045	4.905	7.364	2.459
45.000	0.318	0.075	8.878	0.038	0.033	8.950	10.569	1.619
45.000	2.000	0.033	8.878	0.243	0.015	9.136	11.547	2.411
45.000	2.000	0.075	8.878	0.243	0.033	9.155	11.552	2.397
45.000	2.000	0.117	8.878	0.243	0.052	9.173	11.630	2.457
45.000	3.682	0.075	8.878	0.455	0.033	9.367	12.989	3.623
55.000	1.000	0.050	16.101	0.120	0.022	16.244	18.305	2.061
55.000	1.000	0.100	16.101	0.120	0.045	16.266	18.589	2.323
55.000	3.000	0.050	16.101	0.368	0.022	16.492	21.121	4.630
55.000	3.000	0.100	16.101	0.368	0.045	16.514	20.798	4.284
61.818	2.000	0.075	23.403	0.243	0.033	23.680	27.932	4.253

$\pi_{mix}=\pi_{fructose+CaLac+NaCl}$; $\pi_{addition}=\pi_{fructose}+\pi_{CaLac}+\pi_{NaCl}.$

## Data Availability

The data presented in this study are available on request from the corresponding author.
